# CLAD: A corpus-derived Chinese Lexical Association Database

**DOI:** 10.3758/s13428-019-01208-2

**Published:** 2019-08-19

**Authors:** Shu-Yen Lin, Hsueh-Chih Chen, Tao-Hsing Chang, Wei-En Lee, Yao-Ting Sung

**Affiliations:** 1 Chinese Language and Technology Center, National Taiwan Normal University, Taipei, Taiwan; 2 Department of Educational Psychology and Counseling/Chinese Language and Technology Center/Institute for Research Excellence in Learning Sciences, National Taiwan Normal University, Taipei, Taiwan; 3 Department of Computer Science & Information Engineering, National Kaohsiung University of Science and Technology, Kaohsiung, Taiwan; 4 Graduate Institute of Information and Computer Education, National Taiwan Normal University, Taipei, Taiwan; 5 Department of Educational Psychology and Counseling/Chinese Language and Technology Center/Institute for Research Excellence in Learning Sciences, National Taiwan Normal University, Taipei, Taiwan

**Keywords:** Word association, Lexical association, Association measures, Word co-occurrence, Corpus-derived, Corpus-based, Chinese text corpora

## Abstract

The application of word associations has become increasingly widespread. However, the association norms produced by traditional free association tests tend not to exceed 10,000 stimulus words, making the number of associated words too small to be representative of the overall language. In this study we used text corpora totaling over 400 million Chinese words, along with a multitude of association measures, to automatically construct a Chinese Lexical Association Database (CLAD) comprising the lexical association of over 80,000 words. Comparison of the CLAD with a database of traditional Chinese word association norms shows that word associations extracted from large text corpora are similar in strength to those elicited from free association tests but contain a much greater number of associative word pairs. Additionally, the relatively small numbers of participants involved in the creation of traditional norms result in relatively coarse scales of association measurement, whereas the differentiation of association strengths is greatly enhanced in the CLAD. The CLAD provides researchers with a great supplement to traditional word association norms. A query website at www.chinesereadability.net/LexicalAssociation/CLAD/ affords access to the database.

Words are represented in memory as groups in associative structures, bound together through the specification of values along semantic and episodic dimensions (Masson, 1995; McRae & Boisvert, 1998; Seidenberg, Waters, Sanders, & Langer, 1984). Many scholars have demonstrated different associative strengths between words through a variety of experiments, notably word priming. For example, subjects respond to *nurse* faster than they normally would if it follows a highly associated word such as *doctor* (Meyer & Schvaneveldt, 1971).

Word association has become one of the most common methods of exploring cognitive structures (Fazio, 2001; Plaut & Booth, 2000; Preece, 1976). One frequently used method to obtain word association data is to run free association tests in which a series of stimulus words are presented to respondents who must quickly reply with the word that first comes to mind (the response) upon reading or listening to the stimulus. The underlying assumption of this form of word association test is that stimulus–response relations reflect the structure of words and concepts in the long-term memory. Differences between individuals in lexical associations can thus be used to reveal characteristics about people, such as personality, thinking patterns, affective structure, and so forth (Bargh, Chen, & Burrows, 1996; Crossley, Salsbury, & McNamara, 2015; Greenwald, McGhee, & Schwartz, 1998; Merten & Fischer, 1999; Wu & Chen, 2017).

Beyond using word association to examine individual cognitive structures and processes, psychologists have also investigated the normality of word associations in order to capture the shared relational representations in lexical memory. Several researchers have collected word associations from word association tests and constructed word association norms (De Deyne & Storms, 2008; Jenkins, 1970; Kiss, Armstrong, Milroy, & Piper, 1973; Nelson, McEvoy, & Schreiber, 1998, 2004; Palermo & Jenkins, 1964).

The importance of word association norms is mostly twofold: First, through word associations, researchers can control or manipulate the associative strength of words and precisely select the vocabulary they wish to study (e.g., Nelson, McKinney, Gee, & Janczura, 1998; Siyanova-Chanturia, Conklin, & Van Heuven, 2011). Second, the calculation of lexical relevance has become an important part of language technology and has been widely used in typo detection and correction, automatic text summarization, word sense disambiguation, topic shift detection, and other language processing tasks (e.g., Matsuo & Ishizuka, 2004; Netzer, Feldman, Goldenberg, & Fresko, 2012; Sung, Chang, Lin, Hsieh, & Chang, 2016; Tseng, Chen, Chang, & Sung, 2019). Currently we are likely unable to fully imagine the scope of applications for word association, and new applications are being explored all the time. For instance, Li, Schloss, and Follmer (2017) showed that word association is a good predictor of political party affiliation. Roininen, Arvola, and Lähteenmäki (2006) discovered that word association is a quick and effective way to collect local consumer food preferences. Word association clearly has commercial and social value, and new applications are likely to continue to be put forward.

## The Chen Chinese word association norms

The Chen norms are the largest and most widely used Chinese set of word association norms at present (Hu et al., 2017; Huang, Chen, Huang, & Liu, 2009; Huang, Chen, & Liu, 2012). Hsueh-Chih Chen and his colleagues first selected 900 high-frequency and 900 low-frequency two-character words that are nouns, verbs, adjectives, or adverbs from the *Concise Mandarin Chinese Dictionary* (http://language.moe.gov.tw/index.aspx). Low-frequency words here refer to words with below-average frequencies, whereas high-frequency words refer to the most frequent 13% of words. A preliminary study was conducted to rate the imageability of the words—that is, the extent to which a word evokes a mental image. After one-third of the words with more inconsistent ratings were removed, the imageabilities of the remaining 1,200 words were quite evenly distributed. Then, 1,417 18- to-22-year-old Taiwanese university students enrolled in Introduction to Psychology courses participated in a free association test. Each participant read 200 stimulus words from a paper test book and was tasked with writing down the first word that came to mind. A total of 217 participants whose responses were deemed inappropriate or incomplete were deleted. For each stimulus word, the norms thus collected responses from 200 different participants. We have constructed a query system for the Chen norms, at www.chinesereadability.net/LexicalAssociation/Norm/.

## Limitations of conventional word association norms

Unfortunately, conventional word association norms have limitations that need to be overcome for wide and large-scale applications to be possible. First, word association tests have traditionally only examined a few thousand words, leaving the associative structure of many words unexamined. Furthermore, in past norm construction, despite great effort to recruit thousands of participants usually each participant responded to only one or a few hundred stimuli (Kiss et al., 1973; Nelson et al., 1998, 2004). Thus, the average stimulus word typically received only one to two hundred responses, resulting in a relatively small number of associated words and associative strength measurements that could be more precise. Another problem with word association tests is that the associations of the secondary senses of a word can be suppressed by the primary sense in the free association process. For example, in the study of Kiss et al. the stimulus word *table* only invoked responses associated with the sense of a piece of furniture. However, *table* also commonly refers to a method of displaying information. Words like *figure* and *appendix*, which were not elicited by Kiss et al., would be very likely to be conjured by the reader or listener when this sense of *table* was used in context.

Considering the limitations of using associative norms, we think it necessary to construct a comprehensive (including all common words) and representative (gathered from the natural linguistic productions of a substantial number of people) word association database that includes associations with a wide variety of word meanings. Besides eliciting real-time responses, the use of text corpora may be the most convenient method of obtaining large quantities of linguistic and cognitive information. Corpus-based studies have for many years been used in language-related research, including data mining (Aggarwal & Zhai, 2012), pedagogical and specialized lexicography (Kilgarriff & Grefenstette, 2003), machine translation and learning (Liu, Hsaio, Lee, Chang, & Kuo, 2016; Rauf & Schwenk, 2011; Sung et al., 2015), artificial intelligence (Boden, 1998; McNamara, Crossley, & Roscoe, 2013), and numerous other examples (Chen, Liu, Chen, Wang, & Chen, 2016; Lee, Juan, Tseng, Chen, & Tseng, 2015). Because the linkages between words represent the relationships between the concepts embodied in human language (Li & Zhao, 2017), the numerous connections between words in a large corpus when distilled into an association database could be a great supplement to traditional association norms. We aimed to produce exactly such a corpus-derived Chinese Lexical Association Database (CLAD).

## Computing lexical association strength using word (co-)occurrence frequency data

### Word co-occurrence and lexical association

Deese (1966) stated that “almost all the basic propositions of current association theory derive from the sequential nature of events in human experience” (p. 1). This property arises from the fact that experience is not accumulated from random events. Thus, unlike a lottery ticket, where the next winning number cannot be predicted from the last winner, the percent chance that any given word will appear is altered by what other words have already appeared. For instance, because *apple* is essential to the telling of the famous fairy tale *Snow White*, and because *red* is one of the intrinsic properties of most apples, these words will frequently appear in the same text. Although some words are logically or practically related, others have more idiosyncratic connections. When expressing the idea that tea is highly concentrated, English speakers use the phrase *strong tea*. Although *powerful* is very close in meaning to *strong*, native speakers consistently think that the phrase *powerful tea* is odd (Halliday, 1966). Similar examples are numerous in which the intuitive perception of a word’s tighter relation with one word than another cannot be accounted for on pure syntactic or semantic grounds. It seems that a very good reason to explain the native sense of many fixed lexical usages is a sufficient exposure to the combination (i.e., co-occurrence) of lexical items in text. To summarize, whether reinforced by life experience, natural properties, or lexical idiosyncrasy, these examples show that the co-occurrence of words in text can be used to index lexical association.

Spence and Owens (1990) tested the relation between lexical association and word co-occurrence. They first drew from Palermo and Jenkins’s (1964) word association norms for concrete noun stimuli. For each stimulus, its most common concrete noun response was selected to form an experimental word pair. Then they perused the one-million-word Brown Corpus (Kučera & Francis, 1967) for other concrete nouns that appeared in the corpus with the same frequencies as the response words. These equal-frequency but unrelated words were matched with the stimulus words to generate control pairs. It was found that words of the experimental pairs co-occurred much more frequently in the Brown Corpus than the words of the control pairs. The strong, positive correlation between association rates in the norm and co-occurrence rates in the corpus supports the role of lexical co-occurrence in lexical association. Similarly, Charles and Miller (1989) put forth the hypothesis that “the cue for learning to associate direct antonyms is not their substitutability, but rather their relatively frequent co-occurrence in the same sentence” (p. 357), which was subsequently supported by Justeson and Katz (1991) through their analysis of empirical data.

However, by employing the co-occurrence-oriented approach to retrieve lexical association data, we do not mean to claim that repeated co-occurrence of word forms is a way (or even the only way) to build up lexical association into the structure of our mental lexicon. For the purpose of the present study, we focus on extracting lexical association from text corpora. It is not our goal here to prove whether, or to what extent, association bonds cause co-occurrence or vice versa.

It is nonetheless relevant to ask whether the information retrieved from text corpora based on word co-occurrence is always word associations. The inquiry led us to ponder the definition of word association. McRae and Boisvert (1998) noted that “researchers have typically circumvented the definitional problem [of word association] by operationalizing associative relatedness in terms of word association norms” (p. 569). Given that the current association norms are all restricted in size, the operationalized definition has a fundamental flaw: If a word is not found associated with another word in the norms, one can always argue that the set of norms is simply not large enough. But then again, would corpus-derived word association data differ systematically from traditional norms for which subject recruitment was unlimited? Some recent research has provided a tentative affirmative answer: In four word-priming experiments, Hare, Jones, Thomson, Kelly, and McRae (2009) discovered that certain priming effects can be more adequately explained by event knowledge than by normative word associations. They proposed that nouns should tend to prime other nouns present in related events (e.g., *sale*–*shopper*, *barn*–*hay*, *key*–*door*), and concluded that event-based word relationships are encoded in semantic memory and construed as part of word meaning. Although discussing a more general framework, McRae, Khalkhali, and Hare (2012) commented that “association proper is learning-based; word association [in the operationalized sense] is retrieval or production-based” (p. 45). In line with this argument, we point out that learning may occur implicitly (Frensch & Rünger, 2003), whereas the process of retrieval is mainly explicit. More importantly, what is implicitly learned, including lexical association, may not always be able to be retrieved explicitly, and we would like our corpus-derived lexical association data to fill this gap.

### Co-occurrence window

Some associated words, such as *strong tea*, are almost always adjacent words, whereas other associative words—for instance, *Snow White* and *apple*—are often separated by some number of words. Technically, whether two words can be said to co-occur depends on the distance between them (i.e., the number of intervening words). Studies of word co-occurrence thus often define a specific segment of consecutive text words, known as the *co-occurrence window* or simply *window* (Sinclair, 1991), within which both words occur simultaneously.

### Lexical association measures

Lexical association measures are methods for computing association strengths between words on the basis of their (co-)occurrences in text corpora. Many measures originate in statistical sampling that determines the degree of association by calculating how strongly words co-occur more often than expected by chance (Manning & Schütze, 1999). Some measures compute the entropy of the immediate context of the words by assuming that words occur as units in an information-theoretically noisy environment (Cover & Thomas, 1991; Krenn, 2000). Other measures compute the cosine or dice similarity score of the words based on a vector space model (Frakes & Baeza-Yates, 1992).

Pecina (2010) offered one of the most comprehensive lists of lexical association measures based on the three approaches sketched above. The study also evaluated the performance of the association measures in identifying human-rated associative pairs. Measures of the second and third approaches generally fell into the lower half of performance ranks, while many of the statistically oriented measures performed equally well, topping the rank list. Other relevant studies on word association have also generally relied on statistical measures (Chung & Lee, 2001; Evert & Krenn, 2005; Michelbacher, Evert, & Schütze, 2011; Petrović, Šnajder, & Bašić, 2010). To construct our lexical association database we also used statistical association measures.

The tacit premise that underlies all the statistical measures is the thinking that word association is a hidden parameter that is reflected by the word (co-)occurrence frequencies. We have already pointed out the importance of co-occurrence frequency for word associations. That the frequencies of words occurring individually can also be essential indicators of word association can easily be comprehended. Suppose that word A has identical co-occurrence frequencies with words B and C. However, B’s individual frequency is much higher than C’s. Consequently, A may just be one of many not-so-relevant words for B, whereas it may be a strong associate for C.

The formulas of the 55 statistical measures given in Pecina (2010) are relisted in the Appendix of this article. The statistical association measures all employ part or all of the frequency data, as displayed in a contingency table (see Table 1).

**Table 1:** Contingency table of observed frequencies of (co-)occurrences of a word pair

*a* = *f* (*xy*)	*b* = *f* ($x\overline{y}$)	*f* (x*)
*c* = *f* ($\overline{x}$*y*)	*d* = *f* ($\overline{x}\overline{y}$)	*f* ($\overline{x}$*)
*f* (*y)	*f* (*$\overline{y}$)	*N*

*f *(*xy*) = number of times word *x* and word *y* co-occur. *f *($x\overline{y}$) = number of times that word *x* occurs, and word *y* does not ($\overline{y}$ = any word except *y*). *f *(x*) = sum of *f *(*xy*) and *f *($x\overline{y}$)—that is, the occurrence frequency of *x* (* = any word). *N* = size of the corpus.

### Directionality of word association measures

If one word strongly elicits another while the elicitation in the other direction is relatively weak, the two words are asymmetrically associated. For example, Michelbacher, Evert, and Schütze (2011) showed that in the University of South Florida Association norms (Nelson et al., 1998) the pairs *bird* and *canary* are asymmetrically related: 69% of subjects give *bird* as a response to *canary*, but only 6% give *canary* as a response for *bird*. By contrast, symmetrical association is mutual, that is, it tends to be equally strong in both directions. A good example of symmetric association is *good* and *bad*: The percentages of the subjects give *good* as a response for *bad* and the other way around are 75% and 76%, respectively.

Nearly all corpus-based statistical association measures are nondirectional. In the present study, we have confined ourselves mainly to the symmetrical measures. Among the association measures applied in the CLAD, only two measures—conditional probability and reverse conditional probability—yield asymmetric association strengths for word pairs. The directionality of word association has received increasing attention in recent years. Some recent studies address the importance of investigating asymmetry in word association and/or introduce directional association measures (e.g., Gries, 2013; Hutchison, Heap, Neely, & Thomas, 2014; Michelbacher et al., 2011). Currently, there are only a few asymmetrical association measures, and their abilities to account for behavioral data have been mixed (Gries & Ellis, 2015). In view of the theoretically more precise computation that directional measures can bring about, it is hoped that a sufficient number of asymmetrical measures will emerge that we can test in the near future.

## Approaches to the evaluation of word association measures

The success of extracting word association data from text corpora hinges on the effectiveness of word association measures. Budanitsky and Hirst (2006) noted three kinds of approaches to the evaluation of word association measures. The first kind is a theoretical approach that attempts to optimize preferred mathematical properties. They believed that theoretical evaluations have some uses, but are rather limited in providing evidence as to which measure is superior over another and to what extent. In another approach, association measures are evaluated with respect to their performance in the framework of a particular application with standard references (e.g., typo detection and correction). If some system in natural language processing (NLP), artificial intelligence (AI), or any applied sciences requires measurement of lexical association, different measures can be compared by checking which one makes the system yield the most effective results. Still another approach is based on comparison with human judgments. Setting human judgments as the golden standard gives the assessment of how “good” or “bad” a measure is by its congruence with human performance. These sorts of assessments are currently the most commonly employed (Cramer, Wandmacher, & Waltinger, 2011; Johns & Jones, 2010; Recchia & Jones, 2009).

For this study we employed the third approach to evaluate the word association measures used for construction of the CLAD. We calculated coverage rates of the Chen norms by the CLAD and tested whether the CLAD can predict primed lexical decision latencies nearly as well as (or better than) the Chen norms.

The following article is organized in five sections. The Construction Pipeline of the CLAD section describes how we constructed the CLAD by using natural language processing techniques, including 55 different measures of calculating word associative strength and a 400+-million-word Chinese-language corpus. Next, the How to Use the CLAD section introduces an online query system for using the CLAD. In the How the CLAD Supplements Traditional Association Norms section, we show how the CLAD can supplement traditional association norms. The Comparing the CLAD and the Chen Norms in Predicting Priming Effects section reveals that the CLAD has a greater predictive value than the Chen norms when accounting for primed lexical decision latency. Finally, in the Discussion and Conclusion section, we discuss in a broader context the potential and limitations of automatically constructed word association databases and traditional norms, and make concluding remarks.

## Construction pipeline of the CLAD

**Fig. 1:**
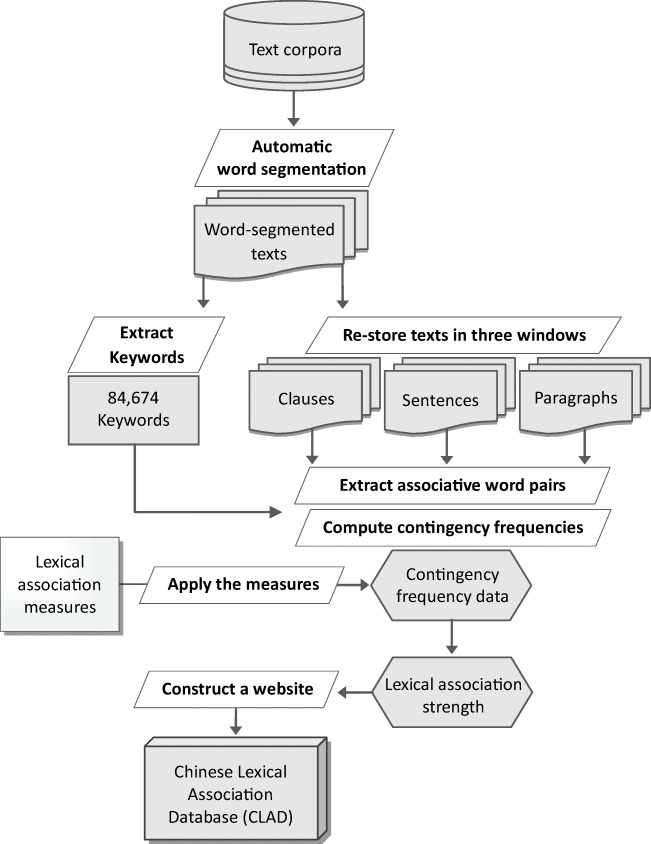
Construction pipeline of the Chinese Lexical Association Database (CLAD)

The automatic construction of a corpus-derived lexical association database involves applying association measures to the word (co-)occurrence data extracted from text corpora. Figure 1 shows the construction pipeline of the CLAD. The various components of the pipeline are described in detail below.

### The text corpora

The various text corpora used for construction of the CLAD are briefly described below. The most substantial portion of the corpora was made up of carefully copyedited texts, to ensure language accuracy. The downside of using published texts is the higher concentration of formal language. To supplement with informal Chinese data, we also used a web-derived corpus. All the texts in each corpus were originally written in Chinese rather than translated to Chinese from another language.

The UDN corpus was collected from three newspapers issued by United Daily News in Taiwan from 2000 to 2012, covering a great variety of theme categories including commentary, culture, education, entertainment, fiction, health, humor, life & style, local news, money & business, people, politics, sports, tech & science, travel, and world news. News on politics, money & business, and sports were the top three contributors. We believe that these theme categories, as perceived by most people, are less dominant in real language use than in the UDN corpus, so we detected these texts through our automatic text classification methods, retained every fourth text, and deleted the others.

The Sinica Corpus was issued by Academia Sinica (http://asbc.iis.sinica.edu.tw/). The collection of texts ranged from 1981 to 2007 covering 14 different text types, including reviews, advertisements or captioned-illustrations, letters, announcements, fiction stories and allegories, prose, biographies and autobiographies, poetry, quotations, manuals, drama scripts, conversations, speeches, and minutes of meetings.

We also utilized a collection of children’s and adolescent books as well as novels for adults. The books for young people were published in recent years and covered life education, stories, and natural science. The publishing dates of the novels ranged from the 1940s to the 2010s, and mostly included science fiction and romance novels.

The only web corpus we used was derived from a bulletin board system called PTT, managed and used mostly by college students in Taiwan (https://www.ptt.cc/index.html). The corpus was drawn from all posts from 2000 to 2016 on the Happy, Sad, Angry, and Hate boards. These web texts were cleaned by removing disqualified context, such as signature files and tag clouds.

The total number of words in the corpora surpasses 400 million; the contribution of each corpus is shown in Table 2.

**Table 2:** Corpora used for the construction of the CLAD and their sizes

Corpus	Number of Word Tokens
United Daily News (UDN) Corpus	253,952,479
Books for children and adolescents	99,340,090
Novels	59,307,704
PTT (a bulletin board system)	10,734,678
Sinica Corpus	9,343,428
Total	432,678,379

### Extracting the keywords

We use the nomenclature of “keyword” and “associate” for the CLAD in the place of “stimulus” and “response,” which are reserved for when referring to association norms. Prior to the extraction of keywords, all texts in the corpora were processed by the Chinese word segmentation and tagging tool developed by Academia Sinica (Tsai & Chen, 2004). A total of 84,674 keywords were extracted by excluding low-frequency, extra-high-frequency, and certain nongeneral words that fall into the criteria below.

1. Words with frequencies below 100.

2. Functional words—that is, words of the five categories: conjunctions, exclamations, prepositions, particles, and pronouns.

3. The 100 highest-frequency words. Most of these words are also function words.

4. Proper nouns with frequencies lower than 500.

5. Time words and quantity words in which numerical characters were more frequent than nonnumerical characters. Numerical characters refer to the Chinese equivalents of the ten Arabic numerals. For example, 二十日 “the twentieth day of a month” (this three-character string is treated as a single word by most Chinese word segmenters) includes two numerical characters (二 “two” and 十 “ten”) and one nonnumber (日 “day”). Because number characters exceeded nonnumbers, the word was excluded. However, 星期三 “Wednesday” was included, because it contains two nonnumerical characters 星 and 期, which together mean “week,” and only a single number 三 “three.”

### Restoring the corpus texts in three co-occurrence windows

In the field of lexicography, whose goal is to pinpoint fixed expressions (also called multiword expressions or collocations) such as *black box* and *roll in*, window size is typically set to five or six words (Church & Hanks, 1990; Smadja, 1993). On the one hand, inquiries of lexical association in the broader, psycholinguistic sense as discussed herein have a greater need to highlight semantic relationships, such as *doctor*/*nurse* and *doctor*/*health*, that often span over larger scales of text words. On the other hand, window size must be limited. Spence and Owens’s (1990) study revealed that when window size exceeds 200 words, co-occurrence rate loses its power to explain word association. Yet it would be wise to also make use of the textual structures pre-defined by language users, namely, the segments delimited by punctuation marks. In the end, we decided to use three co-occurrence windows—clauses, sentences, and paragraphs. By computing lexical association under different windows, we hope that the CLAD can satisfy the needs of a greater variety of users. The three windows are described in detail below:

A paragraph is a string of words delimited by a line break. Like most languages, paragraphs in Chinese indicate how subtopics are organized throughout the text. Its words should be more related among each other than they are related to the words in other paragraphs.

A sentence is separated by any two of the following punctuation marks—periods, question marks, exclamation marks, or semicolons. Unlike the English sentence, which is primarily a grammatical unit, a Chinese sentence is a discursive or rhetoric unit that performs a coherent communicative function. When translated into English, typically a Chinese sentence turns into multiple English sentences.

A clause is framed by two commas or a comma and another punctuation mark. Chinese commas differ greatly from their English equivalents: Though Chinese word strings separated by commas are sometimes similar to clauses in English, they are often more similar to the English sentence unit.

Table 3 gives the total amounts of clauses, sentences, and paragraphs in the corpora. It also shows that the three linguistically defined windows are roughly constrained by size. The mean length of a clause is 6.5 words. On average, a sentence is about four times as long as a clause, and a paragraph is nearly four times the size of a sentence.

**Table 3:** Total number and mean size for each co-occurrence window type

Window type	Total number	Size in Words	
		Mean	*SD*
Clause	68,559,948	6.5	4.9
Sentence	17,007,069	26.3	21.7
Paragraph	4,310,468	96.0	129.6

A total of about 7% of the corpora lack paragraph information and were excluded from the construction of the paragraphs.

### Extracting the associative word pairs and their contingency frequency data

Regardless of sequence, any two words that appeared within a window were treated as co-occurring. For instance, a clause consisting of four different words would generate six word pairs, as illustrated below.

Original clause: 計算詞彙聯想強度 “compute word association strength”

Word-segmented clause: 計算 詞彙 聯想 強度 “compute,” “word,” “association,” “strength”

Word pairs: 計算 詞彙 “compute” “word”

計算 聯想 “compute” “association”

計算 強度 “compute” “strength”

詞彙 聯想 “word” “association”

詞彙 強度 “word” “strength”

聯想 強度 “association” “strength”

We then computed the co-occurring frequencies of those word pairs that were composed of the earlier extracted 84,674 words. Frequencies were defined in terms of how many times a word pair occurred in the clauses, sentences, or paragraphs of the corpora. Say the hypothetical word pair 計算+詞彙 also appeared in 1,000 other clauses, its clause window frequency would be 1,001.

One of the most serious weaknesses of co-occurrence-based lexical association measures is the tendency to obtain unstable results when the co-occurrence frequency is very small (Church & Hanks, 1990). To filter out low-frequency word pairs, we set a relative frequency threshold of *z*-score, followed by a raw frequency limit: The co-occurrence frequencies between a keyword and its co-occurring words were first transformed into *z*-scores. Word pairs with *z*-scores less than 0 were then deleted. Word pairs whose *z*-scores were higher than 0, but raw frequencies were lower than 5, were removed subsequently.

For the remaining word pairs, we computed their contingency frequencies as shown in Table 1. All frequencies were computed using co-occurrence windows as the count units, such that the sizes of the corpora stand for the total numbers of clauses, sentences, or paragraphs in the corpora.

### Computing lexical association strengths and clustering the association measures

We then applied the 55 statistical association measures listed in the Appendix to these contingency frequency data to derive the word association strengths of the associative word pairs.

**Fig. 2:**
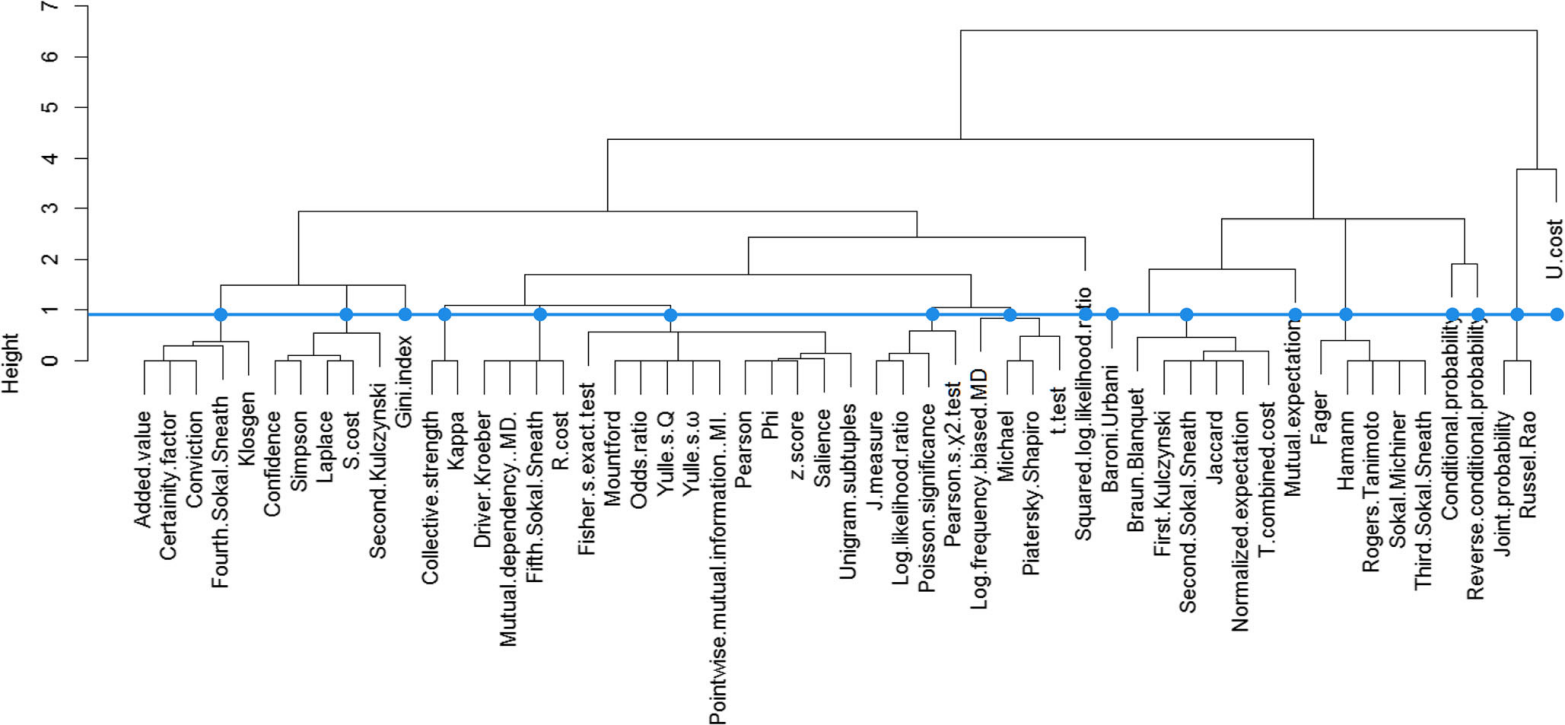
Dendrogram of the word association measures applied to create the CLAD

Tan, Kumar, and Srivastava (2004) demonstrated two theoretical scenarios in which many statistical association measures become consistent with each other. Their theoretical argument was borne out by our empirical study, which revealed that the association strengths yielded by many measures were highly correlated. The analysis we carried out was hierarchical divisive clustering, also commonly known as DIANA (Kaufman & Rousseeuw, 1990). The clustering results can be visualized by a dendrogram (see Fig. 2). Association measures conjoined at a lower branching node have stronger correlations with each other, with the distance metric between cluster members (i.e., the height) being smaller. The number of resultant clusters depends on the threshold imposed on the distance metric. We chose a relatively strict threshold, manifested by the blue line, to ensure great within-cluster similarity. Each vertical line that the blue line crosses represents a group of measures that was identified as a cluster. Accordingly, the measures were divided into 17 clusters.

We calculated the Spearman coefficients between the measures in each cluster. Nearly all the coefficients were higher than .96. To avoid including redundant data, the CLAD presents each cluster by the measure having the highest average coefficient with the other measures in the cluster. The 17 clusters of association measures were represented by the following measures: added value, Baroni-Urbani, conditional probability, Gini index, Jaccard, joint probability, kappa, log likelihood ratio, Michael, mutual expectation, R cost, reverse conditional probability, Simpson, Sokal–Michener, squared log likelihood ratio, U cost, and unigram subtuples.

### Constructing a query website

The CLAD contains the occurrence frequency and part-of-speech (POS) data of the associative word pairs and their association strengths. These were computed by the 17 representative association measures on the basis of the 400+-million-word Chinese-language corpus. A website (www.chinesereadability.net/LexicalAssociation/CLAD/) for querying and downloading these data was constructed.

## How to use the CLAD

### View the whole word list

**Fig. 3:**
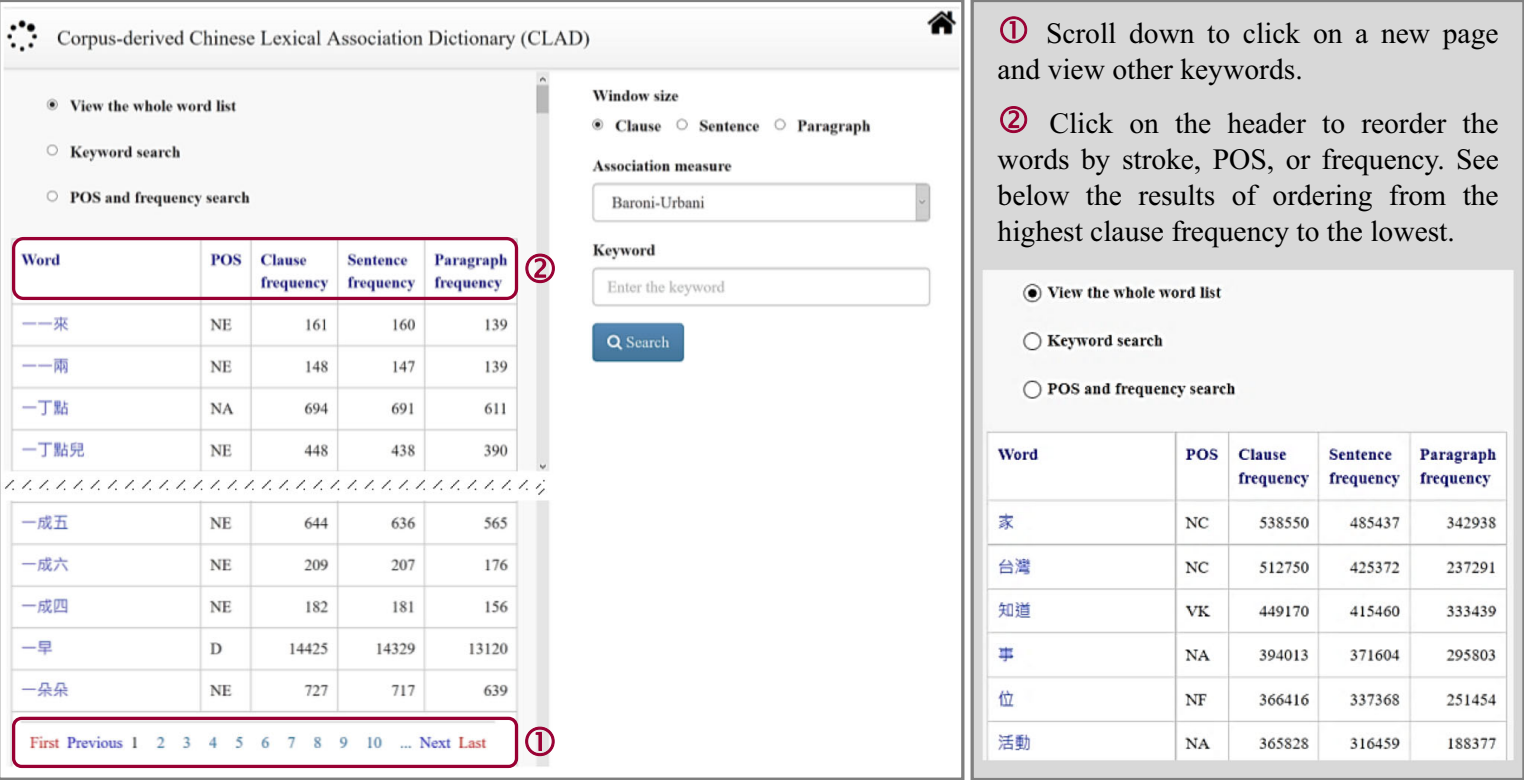
Viewing the whole word list of the CLAD

The “View the whole word list” option allows the user to access all available keywords, 100 per page (see Fig. 3). The most prominent POS (i.e., the most frequently tagged part of speech) and frequency (i.e., number of occurrences in clauses, sentences, or paragraphs in the corpora) of each keyword are also displayed. The keywords are sorted by default from the smallest stroke number to the largest. Clicking on the “Word” header will show the words in the reversed order. If the user clicks on “POS” or “Frequency,” the list will be sorted in accordance with those criteria instead.

### Keyword search

**Fig. 4:**
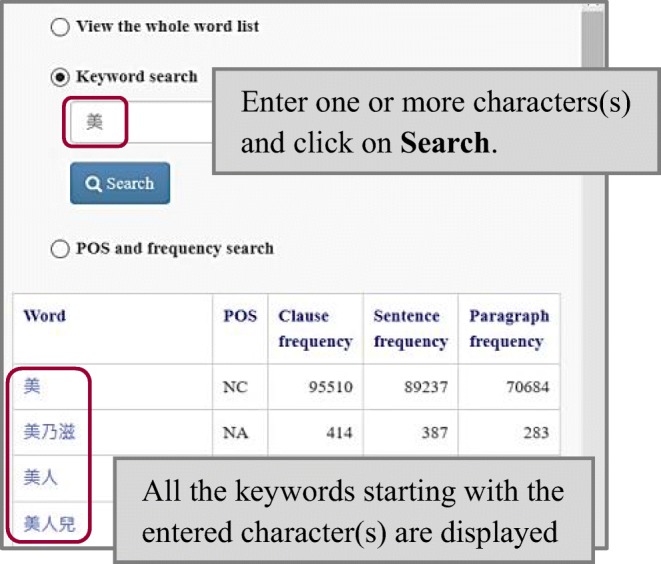
Using the keyword search function

Upon the user clicking the “Keyword search” radio button, a dialog box opens below it. Users can input one or more character(s) into the box to display all the words that start with the entered character(s). This function is exemplified with the character 美 “beautiful” in Fig. 4.

### POS and frequency search

**Fig. 5:**
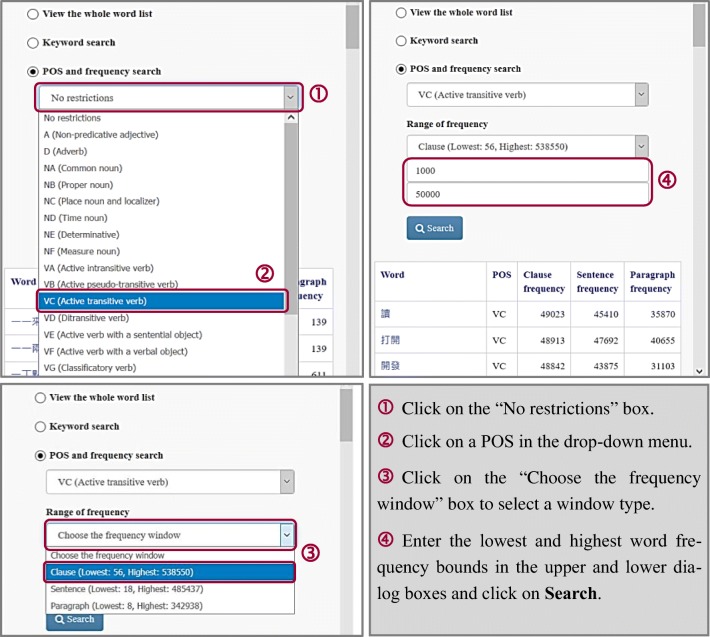
Using the part-of-speech (POS) and frequency search function

Upon clicking the “POS and frequency search” option, users can search for words of a particular POS and/or within a specific frequency range (see Fig. 5). The default setting for the POS option includes all POSs (i.e., “No restrictions”). To browse the vocabulary of a specific POS, first click on “No restrictions.” Next, click on the desired POS in the drop-down menu. Each POS code is followed by a gloss.

To specify the frequency range of keywords, the user must first choose a window in the drop-down menu of “Choose the frequency window.” The lowest and highest word frequencies under each window type are given in the captions. The user can then enter in the two dialog boxes underneath “Range of frequency” the upper and lower frequency bounds of the keywords to be searched for. Notice that the frequency window only pertains to how often the words occur in a given window size. To specify the co-occurrence window under which association strengths were computed, the user must do another selection, to be described below.

### Displaying association data

**Fig. 6:**
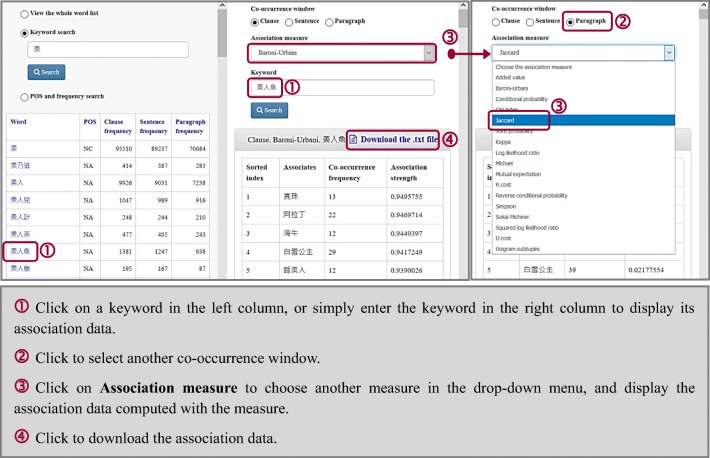
Selecting the co-occurrence window and association measure to display and download association data

Clicking on any of the keywords shown on the left column, such as 美人魚 “mermaid,” prompts the system to display in the right column its associates, as well as the co-occurrence frequencies and association strengths with the associates (see Fig. 6). In addition to using the left column to select a keyword, the user can directly input the word in the “Keyword” dialog box in the right column.

### Selecting co-occurrence window and association measure

Users can choose the desired co-occurrence window of association by clicking the corresponding button for “Clause,” “Sentence,” or “Paragraph” (see Fig. 6). Click on “Association measure” to select from the 17 measures in the resultant drop-down menu.

### Downloading the association data

Click on “Download the .txt file” at the top of the associates table to download the table to the computer (see Fig. 6). The user can drag the icon of the downloaded plain text file to a spreadsheet editor such as Microsoft Excel for optimizing visual display or for further analysis.

## How the CLAD supplements traditional association norms

### Enlarging the size of lexical association data

The Chen norms described above is the largest set of Chinese word association norms at present. The CLAD provides a greater wealth of lexical association data than the Chen norms in terms of both the total number of associative word pairs and the average number of associates for each word.

In terms of the overall size, the Chen norms include about 100,000 word pairs, whereas the CLAD consists of nearly 17 million word pairs using the clause window, more than 165 times that of the Chen norms. When using the sentence or the paragraph window, the CLAD is 644 or 2,015 times larger than the Chen norms, respectively.

For each stimulus word, the Chen norms elicited 86 different response words on average. Under the clause window, the CLAD has, on average, 401 different associates for each keyword, more than four times the number of the Chen norms. Using the sentence or paragraph window, the average increases to 1,523 or 4,516 associates for a keyword, more than 17 or 52 times, respectively, the number for the Chen norms (see Table 4).

**Table 4:** Sizes of the Chen norms and the CLAD

Database	Number of Keywords/Stimuli	Number of Associative Word Pairs	Number of Associates/Responses per Word	
			Mean	*SD*
CLAD				
Clause window	84,674	16,997,424	401	1,338
Sentence window	84,674	66,313,915	1,523	3,569
Paragraph window	84,674	207,538,494	4,516	6,889
Chen norms	1,200	103,006	86	17

### Increasing the differentiation of association strengths

The relatively small numbers of participants involved in the creation of traditional norms have led to associative strength measurements that could be more precise. For an average stimulus in the Chen norms, 68.8% of the responses had a frequency of 1 (i.e., only one participant responded with that particular word to that stimulus). Normative association strengths are computed by dividing the number of particular responses with the number of respondents. Since the number of respondents is the same for every stimulus word in the Chen norms, the association strengths of these once-occurring stimulus–response pairs are identical. In other words, the normative statistics suggests the counterintuitive notion that the majority of associated words are bound with very similar strengths.

In contrast, words in the CLAD are related to the large majority of their associates with different strengths. An average of 98% of the associates of a word can be differentiated by unique association strengths if applying Baroni-Urbani or 11 other association measures. Unique association strengths make up 94% of the associations of an average word for the measures Sokal–Michener and U cost. In contrast to the highly unique strengths of those measures, the average percentages of unique strengths for the other three measures—Simpson, conditional probability, and joint probability—ranged from 8% to 15%.

### Covering the traditional word association norms

For the CLAD to be a good supplement to traditional norms, it should not only provide a greater number of associative data, but also cover the traditional norms (here, the Chen norms). Coverage of the Chen norms can be assessed by discovering how many responses are given with corresponding association strengths in the CLAD. However, the CLAD contains a substantially greater number of associative pairs with considerably higher associative strength differentiation, so it does not seem adequate to assess coverage in a word-for-word fashion such as by computing their correlation coefficients. Considering that responses occurring more frequently in the Chen norms should have relatively, but not exactly, higher association strengths in the CLAD, we utilized the summed relative frequency (SRF) to compute the coverage rate.

#### Computing coverage rate

Taking the stimulus 音樂 “music” as an example, it is described below how to assess whether the responses of an individual stimulus in the Chen norms were covered by the CLAD. Coverage of the norms was then calculated by averaging the coverage rates of the stimuli.

In the Chen norms, the most frequently elicited response to 音樂 “music” was 古典 “classical,” totaling ten tokens (i.e., there were ten participants who responded with 古典 “classical” to 音樂 “music”). The response 欣賞 “to enjoy, to appreciate” had the second highest frequency of nine, followed by the responses 美妙 “pleasant, wonderful” and 歌手 “singer,” both occurring eight times. To compute the SRF of a response, we first summed the frequencies of both the response and the other responses of higher response frequency. The summed frequency was then divided by the total number of response tokens of the stimulus, which was always 200. Accordingly, the SRFs of 古典 “classical” and 欣賞 “to enjoy, to appreciate” were 10/200 = 5% and (9+10)/200 = 9.5%, respectively. 美妙 “pleasant, wonderful” and 歌手 “singer” occurred equally frequently, so they had the same SRF of (8+8+9+10)/200 = 17.5%. The calculation of SRFs continued for the other response words in the same way.

These SRFs were then mapped onto the associates of 音樂 “music” in the CLAD. Coverage of 古典 “classical” was affirmed if it was one of the associates with the highest 5% of association strengths in the CLAD. Suppose 音樂 “music” has 100 associates in the CLAD, then 古典 “classical” must be among the five associates (100×5% = 5) with the highest associative strengths in order to be deemed to be covered. Coverage was negated if 古典 “classical” was not an associate of 音樂 “music,” or if it belonged to the 95% of associates with weaker associative strengths. Likewise, for 欣賞 “to enjoy, to appreciate” or 美妙 “pleasant, wonderful” 歌手 “singer” to be covered, they must be among the strongest 9.5% or 17.5% of the associates in the CLAD. The checking continued for the other responses in the same way. The number of covered responses was divided by the total number of different responses (i.e., response types) to return the coverage rate of 音樂 “music.”

#### Coverage rates computed using associates and stimuli in varying strength and frequency ranges

For the following two reasons, we hold that coverage rates computed using stimuli with different word frequencies and associates of different association strengths (i.e., instead of only using all the associates and all the stimuli) can be used to help evaluate the CLAD more efficiently. We will then make two predictions for a good corpus-derived word association database regarding how coverage rates should change with the variation of word frequencies and associative strengths.

A terminological distinction should be noted at this juncture: We use the term “word frequency” to denote how often a word occurs in the corpora, whereas “response frequency” refers to how frequently a word was elicited in a word association test. High- and low-frequency words are thus the more common and the relatively rare words in the corpora.

Our first reason is based on the fact that in comparison to low-frequency words, high-frequency words possess larger numbers of co-occurring instances in text corpora. In statistical terms, the larger sample reduces the probability of inaccurate computation by the association measure. Accordingly, corpus-derived lexical association data obtained using high-frequency words should enjoy a higher likelihood of reflecting lexical relationships in the mental lexicon. One can therefore make the prediction that the number of normative responses covered by the CLAD should increase when using stimuli with increasing word frequencies.

The second reason is related to the assumption underlying the entire history of free association study; that is, words with stronger cognitive bonds are easier to retrieve, so that more frequently elicited responses possess stronger association strengths in the human mind. One may then reasonably assert that the Chen norms responses should be found possessing relatively high association strengths in the CLAD. To put in another way, on the whole associates in the CLAD that are also responses in the Chen norms should be more strongly associated with the keywords than those associates not found in the Chen norms. Predictably, when we increase the number of tested associates, but lower their association strengths at the same time, coverage rates should increase at a decreasing rate.

To test the two predictions, coverage rates were computed using four sets of associates and four sets of stimuli in varying strength and frequency ranges. Always starting with the associate with the strongest association strength, we picked the top 10%, the top 40%, the top 70%, and the top 100% (i.e., all) of the associates according to their association strengths in the CLAD. As such, with the number of associates increased to four, seven, and ten times larger (i.e., from 10% to 40%, 70%, and 100%), their overall association strengths gradually decreased. Likewise, always starting with the word of the highest frequency, we picked the top 25%, the top 50%, the top 75%, and the top 100% (i.e., all) of the stimuli according to their word frequencies in the corpora, so that the overall word frequencies of the four stimulus sets gradually decreased.

Table 5 presents the coverage rates computed by using the various sets of associates and stimuli under three window sizes for the 17 association measures. To examine the first prediction, we compared the coverage rates based on the four sets of stimuli. (Each comparison was done using the same set of associates to rule out the effect of associative strengths.) The critical rationale underlying our analysis is that if the word frequency of a stimulus does not play a role in the variation of coverage rate, coverage rates obtained using the four frequency-varying sets of stimuli should be identical.^1^ However, if using high-frequency words to construct word association data has a better chance to align with the norms, the highest coverage rate should be yielded by using the stimuli at the highest frequency range of 25%. Using the stimuli at the top 50% of word frequency would yield the second highest coverage rate, and so on and so forth.

**Table 5:** Coverage rates of the Chen norms in the CLAD, computed using stimuli and associates in varying frequency and strength

	Clause Window																Sentence Window																Paragraph Window															
	All				75%				50%				25%				All				75%				50%				25%				All				75%				50%				25%			
	10 %	40 %	70 %	All	10 %	40 %	70 %	All	10 %	40 %	70 %	All	10 %	40 %	70 %	All	10 %	40 %	70 %	All	10 %	40 %	70 %	All	10 %	40 %	70 %	All	10 %	40 %	70 %	All	10 %	40 %	70 %	All	10 %	40 %	70 %	All	10 %	40 %	70 %	All	10 %	40 %	70 %	All
Added value	.12	.30	.38	.42	.16	.39	.49	.55	.21	.50	.63	.70	.23	.55	.70	.78	.23	.46	.54	.59	.30	.57	.68	.73	.36	.68	.80	.86	.36	.70	.83	.90	.30	.55	.66	.71	.37	.66	.78	.84	.41	.74	.86	.92	.38	.73	.86	.93
Baroni-Urbani	.17	.31	.38	.42	.22	.40	.49	.54	.31	.53	.63	.69	.40	.63	.73	.78	.29	.46	.54	.58	.37	.58	.67	.72	.50	.71	.80	.85	.59	.81	.88	.91	.36	.56	.64	.70	.45	.67	.75	.82	.58	.80	.87	.91	.65	.87	.92	.94
Conditional probability	.17	.31	.38	.42	.22	.41	.49	.55	.29	.54	.64	.70	.36	.63	.73	.79	.28	.47	.54	.59	.36	.59	.68	.73	.46	.74	.82	.86	.54	.81	.88	.91	.35	.58	.66	.71	.44	.71	.80	.84	.55	.83	.90	.92	.59	.87	.92	.94
Gini index	.17	.31	.38	.43	.23	.41	.49	.55	.31	.53	.63	.70	.37	.60	.71	.78	.32	.48	.55	.59	.41	.60	.68	.73	.52	.73	.81	.86	.58	.78	.86	.91	.41	.60	.67	.71	.51	.72	.80	.84	.62	.83	.89	.92	.66	.85	.91	.94
Jaccard	.19	.33	.40	.43	.25	.43	.51	.55	.34	.57	.66	.70	.41	.65	.74	.79	.33	.50	.56	.59	.42	.63	.70	.73	.54	.76	.83	.86	.60	.82	.88	.91	.42	.61	.68	.71	.52	.73	.80	.84	.62	.84	.90	.92	.64	.87	.92	.94
Joint probability	.17	.31	.38	.42	.22	.41	.49	.55	.29	.54	.64	.70	.36	.63	.73	.79	.28	.47	.54	.59	.36	.59	.68	.73	.46	.74	.82	.86	.54	.81	.88	.91	.35	.58	.66	.71	.44	.71	.80	.84	.55	.83	.90	.92	.59	.87	.92	.94
Kappa	.19	.33	.39	.43	.25	.42	.50	.55	.34	.56	.64	.70	.42	.64	.72	.78	.33	.49	.55	.59	.43	.62	.69	.73	.55	.75	.81	.86	.62	.81	.86	.91	.43	.61	.68	.71	.53	.73	.80	.84	.64	.84	.89	.92	.66	.87	.92	.94
Log likelihood ratio	.19	.32	.39	.43	.25	.42	.50	.55	.34	.54	.63	.70	.40	.61	.71	.78	.34	.49	.55	.59	.44	.61	.68	.73	.54	.73	.81	.86	.60	.78	.86	.91	.44	.62	.68	**.72**	.54	.74	.80	**.84**	.64	.84	.89	**.92**	.67	.86	.91	**.94**
Michael	.19	.33	.39	.43	.25	.43	.50	.55	.33	.56	.64	.70	.41	.64	.72	.79	.32	.49	.55	.59	.41	.62	.69	.73	.53	.75	.82	.86	.60	.81	.86	.91	.39	.60	.68	.71	.49	.73	.80	.84	.60	.84	.90	.92	.64	.88	.92	.94
Mutual expectation	.19	.33	.40	.43	.25	.43	.51	.55	.33	.56	.65	.70	.40	.65	.74	.79	.32	.50	.56	.59	.41	.63	.70	.73	.51	.75	.83	.86	.57	.82	.88	.91	.42	.62	.68	.72	.50	.74	.81	.84	.59	.84	.90	.92	.61	.87	.92	.94
R cost	.18	.33	.39	.43	.23	.42	.51	.55	.31	.55	.65	.70	.38	.63	.73	.79	.33	.50	.56	.59	.42	.62	.70	.73	.53	.75	.83	.86	.60	.81	.88	.91	.44	.62	.68	.72	.53	.74	.81	.84	.63	.84	.90	.92	.67	.87	.92	.94
Reverse conditional probability	.10	.29	.38	.42	.14	.37	.49	.54	.18	.48	.62	.69	.21	.53	.69	.77	.19	.43	.54	.59	.24	.54	.67	.73	.30	.64	.78	.85	.32	.67	.83	.90	.24	.52	.64	.71	.29	.61	.75	.83	.32	.68	.83	.91	.33	.69	.85	.93
Simpson	.12	.29	.38	.42	.16	.38	.48	.54	.21	.49	.62	.69	.23	.55	.70	.78	.22	.44	.53	.58	.28	.56	.67	.73	.35	.68	.80	.86	.36	.71	.84	.90	.28	.54	.65	.70	.35	.66	.77	.83	.41	.74	.86	.92	.39	.74	.87	.93
Sokal–Michener	.03	.16	.27	.37	.04	.20	.34	.47	.05	.24	.41	.59	.04	.22	.41	.64	.05	.19	.32	.49	.06	.22	.38	.60	.05	.21	.39	.69	.04	.16	.33	.70	.06	.19	.31	.57	.06	.19	.33	.66	.04	.14	.29	.70	.04	.12	.25	.70
Squared log likelihood ratio	.04	.19	.30	.39	.06	.25	.39	.50	.07	.30	.48	.62	.06	.29	.49	.68	.07	.24	.37	.52	.08	.29	.45	.63	.08	.29	.48	.72	.06	.23	.42	.73	.08	.23	.37	.60	.08	.24	.39	.68	.05	.19	.35	.72	.04	.14	.29	.71
U cost	.05	.18	.29	.38	.07	.23	.37	.49	.08	.29	.47	.62	.10	.33	.53	.70	.10	.29	.42	.54	.12	.35	.52	.66	.14	.40	.60	.77	.21	.54	.73	.86	.15	.39	.54	.66	.17	.46	.63	.77	.22	.55	.73	.86	.32	.70	.83	.92
Unigram subtuples	.14	.31	.39	.43	.18	.40	.50	.55	.24	.52	.64	.70	.29	.59	.72	.78	.27	.48	.56	.59	.34	.60	.69	.73	.42	.72	.82	.86	.47	.77	.87	.91	.35	.59	.67	.71	.43	.70	.79	.84	.49	.79	.88	.92	.50	.81	.90	.94

The prediction was confirmed by the vast majority of the comparisons of coverage rates shown in Table 5. For example, for the log likelihood ratio measure, the coverage rate computed using all the associates against all the stimuli under the paragraph window was .72. The rates increased to .84, .92, and .94 when the word frequencies of the tested stimuli increased to the top 75%, 50%, and finally to the highest frequency range of 25% (see the boldfaced rates in Table 5).

The opposite trend, in which higher word frequencies of the stimuli were associated with lower coverage rates, was found in a single comparison for Simpson under the paragraph window using 10% of the associates based on the stimuli of the highest 50% and 25% of word frequencies (the coverage rates were .41 and .39, respectively), in two comparisons for added value, and in several comparisons for Sokal–Michener and squared log likelihood ratio.

To test the second prediction, the coverage rates obtained using 40%, 70%, and 100% of the associates were divided by the coverage rate obtained using the strongest 10% of associates. There were three possibilities regarding the size of the resultant quotients. First, if the covered responses were evenly distributed among the CLAD associates, the only factor that affects the resultant quotients would be the number of associates used for the computation. Therefore, the quotients would be 4, 7, and 10 (40%÷10% = 4, 70%÷10% = 7, 100%÷10% = 10). The average quotient would be 7 [(4+7+10)/3 = 7].

The second possibility, which is demonstrative of our prediction for a good association database, is that association strengths of the normative word pairs are relatively high in the CLAD, such that more than 10% of the normative responses are covered by the strongest 10% of the CLAD associates. Compared with the first possibility, the divisor (i.e., the coverage rate yielded by using the strongest 10% of associates) would become larger, and the resultant quotients would be smaller than 4, 7, and 10, whose average would be smaller than 7. If the association strengths of the normative responses became even stronger in the CLAD, even more responses would be covered by the associates with high association strengths. Consequently, the resultant average quotients would become even smaller than 7.

The third possibility occurs when the normative responses tend to be weaker associates in the CLAD, resulting in average quotients that are larger than 7. Table 6 gives the actual average quotients computed for the 17 association measures. Only two measures, Sokal–Michener and squared log likelihood ratio, returned average quotients larger than 7. The other, much smaller average quotients show that many of the association measures yielded relatively high association strengths for the stimulus–response pairs in the Chen norms.

**Table 6:** Tendencies of the association measures to align with the Chen norms

Measure	Window Type		
	Clause	Sentence	Paragraph
Added value	2.98	2.25	2.11
Baroni-Urbani	2.04	1.65	1.57
Conditional probability	2.13	1.78	1.70
Gini index	2.04	1.59	1.49
Jaccard	1.91	1.57	1.50
Joint probability	2.13	1.78	1.70
Kappa	1.88	1.53	1.46
Log likelihood ratio	1.88	1.51	1.42
Michael	1.93	1.58	1.56
Mutual expectation	1.94	1.62	1.55
R cost	2.05	1.57	1.45
Reverse conditional probability	3.35	2.60	2.53
Simpson	2.96	2.29	2.14
Sokal–Michener	8.24	7.74	7.77
Squared log likelihood ratio	6.96	6.25	7.28
U cost	5.48	4.07	3.23
Unigram subtuples	2.63	1.94	1.80

Smaller values indicate a stronger tendency to align.

The results of testing the two predictions can be used not only to appraise the CLAD on the whole, but also to evaluate the effectiveness of the association measures individually. The first prediction result rendered Simpson, added value, Sokal–Michener, and squared log likelihood ratio less favorable than the other measures. It is probably not coincidental that these four measures also performed less well for the second prediction.

In addition to confirming the CLAD’s alignment with the Chen norms, promoting the corpus-based approach to constructing lexical association data requires that we provide more supportive evidence, such as the explanatory power of the CLAD on human behavioral performance, a topic that we now turn to.

## Comparing the CLAD and the Chen norms in predicting priming effects

Lexical associations derived from both text corpora and free association tests have been identified as an important variable in predicting word-priming effects (Balota & Paul, 1996; Brunellière, Perre, Tran, & Bonnotte, 2017; Günther, Dudschig, & Kaup, 2016; Hutchison, 2003; Lupker, 1984; Shelton & Martin, 1992). We are interested in discovering whether the CLAD might exhibit as much predictive power in human behavior as traditional association norms do. A word-priming experiment using a lexical decision task (LDT) was conducted for this study. In most LDT priming studies, participants are asked to make the “word” versus “nonword” decision upon reading a letter string (i.e., the target) that is preceded by a priming word. If the prime and the target are related, a priming effect is anticipated such that the reaction time would be shorter than if the two words were unrelated.

### Utilizing the Chinese Lexicon Project as a baseline database

There have been recent attempts to compile mega-scale real-time behavioral responses pertaining to lexical items (e.g., the English Lexicon Project; Balota et al., 2007). They provide substantial materials on the basis of which researchers can conduct related studies and test theoretical models. A large database of Chinese lexical decision performance in a neutral (i.e., no priming) condition was published recently for more than 25,000 traditional Chinese two-character words (Chinese Lexicon Project; Tse et al., 2017). In this project, participants were asked to decide whether a two-character string visually presented to them formed a legitimate Chinese word. The reaction time and accuracy data were collected. Past large behavioral databases have been found to be quite robust with respect to relatively large sets of independent variables (Balota, Cortese, Sergent-Marshall, Spieler, & Yap, 2004). The neutral reaction time data utilized in the present priming experiment were derived from the Chinese Lexicon Project.

### The priming experiment

#### Method

##### Participants

A total of 60 native Chinese speakers, 25 males and 35 females, aged between 21 and 43 participated in the experiment. All worked as either research assistants or postdoctoral fellows at National Taiwan Normal University. None of them are on the present research team, and all volunteered to take part in the priming experiment.

##### Materials

In all, 102 related and 102 unrelated prime–target pairs were selected using the following criteria and procedure. First, we extracted all associative word pairs on the basis of both the Chen norms and the CLAD. We used the CLAD data under the paragraph window as it provided a much larger pool than the clause and sentence windows. The words also had to appear in the Chinese Lexicon Project. The qualified word pairs were then grouped according to their association strengths in the Chen norms. In each group, word pairs were sorted by the word frequencies of target words (i.e., the Chen norm responses/CLAD associates). We then selected word pairs at equal intervals of target word frequencies, such that all ranges of word frequencies were well represented. The interval was identical for all the groups (every 200th pair) except for the group of the least strong association (i.e., the associative pairs occurring only once in the Chen norms), whose interval was doubly large (every 400th pair). The percentages of the tested word pairs according to their normative association strengths are given in Table 7.

**Table 7:** Percentages of word pairs in the Chen norms and the priming experiment, according to their normative association strengths

Association Strength in the Chen Norms	Percentage in the Chen Norms	Percentage in the Priming Experiment
≧.03	7.2	15.7
≧.015 & < .03	10.5	26.5
= .01	13.2	21.6
= .005	69.2	36.3

Association strength is calculated by dividing the number of particular responses by the number of respondents, which is always 200 for a stimulus in the Chen norms.

The unrelated word pairs were created by recombining the primes and targets of the related pairs, with the constraint that the paired words were not associated in either the forward or the backward direction in either the Chen norms or the CLAD. We executed a selection algorithm on a computer over 1,000 iterations, and 102 pairings was the maximum number that could be extracted.

A total of 102 nonwords were generated by recombining the distinct characters of the tested words. Three native speakers made the word-versus-nonword judgment without knowing the purpose of the study beforehand. The characters were recombined several times until uniform agreement of nonword was reached by the raters. The words that were paired with the nonwords were different from the primes and targets.

Two lists of testing items were constructed and counterbalanced so that each word occurred only once in a list. Each participant was tested on one of the two lists. As a result, each testing item received responses from 30 participants. Each list was divided into two blocks, with each block consisting of 102 trials (25 or 26 related pairs, 26 or 25 unrelated pairs, and 51 word–nonword pairs). The order of the blocks was also counterbalanced across participants. The order of the word pairs in each block were randomized for each participant.

##### Procedure

The experiment was run on a PC with 19-in. flat-screen display via the E-Prime software (Schneider, Eschman, & Zuccolotto, 2002). The instructions emphasized both speed and accuracy. Prior to the experimental session, participants took a practice session consisting of 20 randomized trials (five related pairs, five unrelated pairs, and ten word–nonword pairs). The displayed words were presented in font size 36. Participants read each two-character word from left to right. Prime and target words were displayed in the font types 新細明體 (PMingLiU) and 標楷體 (DFKai-SB), respectively. Each trial began with a fixation mark (+) appearing in the center of the screen for 500 ms. The prime appeared for 200 ms, followed by a blank screen for 100 ms (stimulus onset asynchrony (SOA) = 300 ms). After the blank, the target appeared, which remained on the screen until the participant pressed the key labeled either 是 (the L key) or 否 (the A key), indicating word and nonword status, respectively. Once they pressed a key, participants received a 200-ms message that informed them whether they gave the right or the wrong answer. The next trial began after a 1,500-ms blank-screen intertrial interval. There was a short break between the two blocks of the experimental session. Participants were asked to determine the length of the break on their own while remaining in their seats.

#### Results

Researchers have demonstrated that *z*-score transformation of raw reaction times in a priming experiment reduces variance across participants (Bush, Hess, & Wolford, 1993; Faust, Balota, Spieler, & Ferraro, 1999), and consequently increases reliability of item-based analysis (Hutchison, Balota, Cortese, & Watson, 2008). The results of the present experiment thus underwent a procedure of standardization that transformed each raw reaction time (henceforth, raw RT) into a *z*-score (*z*RT) based on a participant’s overall RTs. Then we trimmed the results by discarding reactions greater than two standard deviations from the mean. The trimming removed 2.65% of the overall reactions. Applying an intersubject Cronbach’s alpha reliability test to both the raw RTs and the *z*-scores attested the variance-reducing effect of *z*-score transformation: For one testing list, the Cronbach’s alpha coefficients based on the raw RTs and *z*RTs were .62 and .71, respectively. For the other testing list, the coefficients were .72 and .83, respectively.

For a one-way analysis of variance test of the priming effect (i.e., a significant reaction time difference between the related and unrelated word pairs), the *F* value also increased as a result of the *z*-score transformation. For the raw-RT-based priming effect, the mean was 25.51 ms (*SD* = 67.41 ms), *F*(1, 202) = 7.22, *p* < .01. When *z*-scores were used instead, the statistics were *M* = 0.11, *SD* = 0.23, *F*(1, 202) = 9.32, *p* < .01.

#### Multiple regression analyses

The influence of word-related features in semantic priming has been investigated in several studies. In terms of the analytics adopted, these studies fall into two groups (Mandera, Keuleers, & Brysbaert, 2017). The traditional group of factorial design analysis has been shown to contain several potential confounds (Balota et al., 2004; Forster, 2000). For example, there are few, if any, words whose characteristics differ in only one dimension. As a result, the item sets can hardly be matched in the other dimensions across conditions. Researchers’ implicit knowledge may bias their selection of test items, which would then work to achieve their desired effects. Researchers also tend to utilize items that have extreme values across a certain characteristic such that list contexts often vary across experiments. Most importantly, because many of the semantic or psychological features of words are continuous variables, an analysis of the priming effect should indicate its extent, not just its presence or absence (McRae, De Sa, & Seidenberg, 1997).

An alternative methodological approach that could minimize the problems of factorial designs is to model the semantic priming at the item level through regression-based analysis. For example, Hutchison, Balota, Cortese, and Watson (2008) examined 15 predictor variables that modulate the size of the semantic-priming effect through a multiple regression procedure. Using a similar analytical method, we conducted simultaneous multiple regression analyses at item-based level to ascertain the value of various possible predictors in accounting for the variance of the observed priming effect.

##### Predictor variables

The predictor variables entered into the regression model included the lexical and behavioral characteristics of the related primes, the unrelated primes, and the targets, as well as the associative strengths in the Chen norms and the CLAD. Description and summary statistics of the variables are given in Table 8.

**Table 8:** Means, standard deviations, and ranges for the predictor variables used in the regression analyses

Predictor Variables		Mean	*SD*	Range
Prime	Stroke	21.61	6.07	(7, 43)
(related and unrelated)	Word frequency	7.81	1.47	(4.81, 10.65)
	Left orthographic neighbors	59.36	54.10	(0, 298)
	Right orthographic neighbors	102.07	121.31	(1, 654)
	*z*RT_CLP_	– 0.13	0.43	(– 0.87, 1.05)
Target	Stroke	21.48	6.87	(5, 40)
	Word frequency	8.92	1.29	(5.87, 11.87)
	Left orthographic neighbors	80.5	74.61	(2, 289)
	Right orthographic neighbors	102.16	131.35	(2, 654)
	*z*RT_CLP_	– 0.32	0.33	(– 0.95, 1.17)
Associative	Chen Norms			
	Forward	.016324	.016619	(.005, .105)
	Backward	.225938	.280689	(.004, 1)
	CLAD			
	Added value	0.048222	0.076665	(– 9.4e-05, 0.515925)
	Baroni-Urbani	0.551819	0.202924	(0.058325, 0.903283)
	Conditional probability	0.038788	0.057208	(0.000319, 0.449438)
	Gini index	6e-06	1.8e-05	(4.63e-10, 8.8e-05)
	Jaccard	0.00828	0.012235	(9.5e-05, 0.071429)
	Joint probability	2.5e-05	4.6e-05	(1e-06, 0.000274)
	Kappa	0.015398	0.023186	(– 0.000159615, 0.133028)
	Log likelihood ratio	513.248121	1,134.09	(0.180993, 6,932.112495)
	Michael	9.30e-05	0.000177	(2e-06, 0.001083)
	Mutual expectation	1.00e-06	4.00e-06	(4.42e-10, 0.000029)
	R cost	0.002334	0.007047	(3e-06, 0.058224)
	Reverse conditional probability	0.023922	0.060867	(9.6e-05, 0.518809)
	Simpson	0.051452	0.077473	(0.002340824, 0.518809)
	Sokal–Michener	0.996286	0.004341	(0.975672, 0.999796)
	Squared log likelihood ratio	16.441521	30.86232	(0.174048, 201.581215)
	U cost	0.351233	0.263277	(0.002478, 0.979513)
	Unigram subtuples	2.079332	1.534075	(– 1.313466, 6.388045)

Stroke = number of strokes of the two characters of a word. Word frequency = logarithmic transformation (base *e*) of word frequency in the text corpora described in Table 3. Left orthographic neighbors = number of words sharing the same first character. Right orthographic neighbors = number of words sharing the same second character. *z*RT_CLP_ = standardized RT according to the Chinese Lexicon Project. Associative = Chen and CLAD association strengths computed using the various measures. Forward = proportion of the target in the overall responses to the prime in the Chen norms. Backward = proportion of the prime as a stimulus in the overall associations in the Chen norms when the target was a response.

##### Model *R*^2^ and regression coefficients

Because the CLAD variables based on the association measures were entered into the regression models individually, we ran 17 multiple regression analyses in total. Table 9 gives the *R*^2^ values of the models and the standardized regression coefficients, or beta weights, for the variables, based on the related primes, the unrelated primes, and the targets. The row names in Table 9 have the sole function of specifying the regression models into which the referred associative variables were entered. The beta weights for the associative variables themselves are given in Table 10.

**Table 9:** *R*^2^ of the multiple regression models and beta weights for the lexical and behavioral variables predicting the priming effect

	Model *R*^2^	Un Prime Stroke	Un Prime L Ortho	Un Prime R Ortho	Un Prime Freq	Un Prime *z*RT_CLP_	Rel Prime Stroke	Rel Prime L Ortho	Rel Prime R Ortho	Rel Prime Freq	Rel Prime *z*RT_CLP_	Target Stroke	Target L Ortho	Target R Ortho	Target Freq	Target *z*RT_CLP_
Added value	.24	.08	.15	– .01	.06	.14	.01	– .04	– .05	– .15	– .10	.06	– .01	.15	– .33^†^	.12
Baroni-Urbani	.29^*^	.08	.10	– .01	.13	.19	.00	– .05	– .06	– .17	– .11	.07	.02	.16	.02	.23
Conditional probability	.24	.08	.16	.00	.06	.15	.01	– .05	– .06	– .13	– .12	.05	– .02	.15	– .35^†^	.12
Gini index	.26^**†**^	.10	.17	– .03	.09	.16	.00	– .07	– .05	– .23	– .14	.05	– .02	.14	– .31^†^	.14
Jaccard	.29^*^	.11	.13	.00	.18	.25^†^	.02	– .10	– .09	– .27^†^	– .16	.05	– .01	.17	– .21	.17
Joint probability	.28^*^	.11	.16	– .01	.14	.19	.03	– .10	– .06	– .32^*^	– .15	.06	– .01	.15	– .34^†^	.14
Kappa	.29^*^	.11	.13	.00	.18	.25^†^	.02	– .10	– .09	– .27^†^	– .16	.05	– .01	.17	– .21	.17
Log likelihood ratio	.29^*^	.11	.16	– .03	.16	.21	.02	– .10	– .06	– .30^*^	– .16	.04	– .01	.15	– .29	.14
Michael	.28^*^	.11	.16	– .02	.15	.19	.03	– .10	– .06	– .32^*^	– .15	.06	– .01	.15	– .33^†^	.14
Mutual expectation	.29^*^	.10	.18	– .02	.16	.22^†^	.04	– .11	– .08	– .27^†^	– .15	.03	– .01	.15	– .29	.13
R cost	.26^**†**^	.09	.16	– .01	.10	.19	.02	– .06	– .07	– .20	– .14	.05	– .02	.15	– .29	.14
Reverse cond. prob.	.24	.08	.14	– .02	.05	.14	.00	– .04	– .04	– .16	– .08	.08	.00	.15	– .30	.12
Simpson	.24	.08	.15	– .01	.05	.14	.01	– .04	– .05	– .15	– .10	.06	– .01	.15	– .33^†^	.12
Sokal–Michener	.25	.05	.13	– .03	.08	.14	.02	– .05	– .06	– .09	– .09	.06	– .02	.14	– .12	.14
Squared log likelihood	.24	.08	.14	– .01	.04	.14	.01	– .05	– .06	– .08	– .08	.06	– .01	.14	– .27	.12
U cost	.27^*^	.08	.11	.01	.02	.15	.01	– .04	– .07	– .20	– .07	.06	– .03	.12	– .16	.20
Unigram subtuples	.26^**†**^	.09	.12	– .01	.11	.17	– .01	– .05	– .06	– .13	– .10	.06	.01	.17	– .22	.15

Un = unrelated; Rel = related; Freq = frequency; L = left; R = right; Ortho = orthographic neighbors; *z*RT_CLP_ = *z*-score standardized reaction time from the Chinese Lexicon Project (Tse et al., 2017). ^*^p < .05; ^†^*p* < .10.

**Table 10:** Beta weights for the associative variables predicting the priming effect

CLAD		Chen Norms	
		Forward	Backward
Added value	.09	.12	– .01
Baroni-Urbani	.37^*^	– .01	– .01
Conditional probability	.12	.09	– .01
Gini index	.22^**†**^	.07	– .04
Jaccard	.34^*^	– .02	– .03
Joint probability	.30^*^	.05	– .02
Kappa	.34^*^	– .02	– .03
Log likelihood ratio	.32^*^	.02	– .02
Michael	.31^*^	.05	– .02
Mutual expectation	.31^*^	.03	– .01
R cost	.21	.04	– .03
Reverse cond. prob.	.10	.14	– .01
Simpson	.08	.12	– .01
Sokal–Michener	.19	.13	.03
Squared log likelihood	.08	.14	.02
U cost	.27^*^	.16	.03
Unigram subtuples	.21	.06	– .02

^*^p < .05, ^†^*p* < .10.

The regression models based on the following association measures—Baroni-Urbani, Jaccard, joint probability, kappa, log likelihood ratio, Michael, and mutual expectation—were able to account for a larger proportion of the priming variance (*R*^2^ = .28 or .29, all *p*s < .05) than the other models. These association measures also turned out to be the strongest predictors among the variables, with the beta weights reaching between .30 and .37 (all *p*s < .05). The Chen norms did not exhibit significant predictive power in the priming results, with the largest beta weights being only .16 and .03, for forward and backward associations, respectively.

The results of the priming experiment indicate the superiority of the CLAD over the Chen norms in accounting for human performance. The multiple regression analysis also provides users a guide to which measures (i.e., those with larger beta weights) are probably more useful for applications of the CLAD. Moreover, as most association measures are derived from statistical or probabilistic models, the theoretically oriented models (or measures) need to be verified by empirical observations, for which the findings of our study can provide a useful aid. Specifically, the priming results give information on the varying degrees of validity of the association measures in extracting lexical association from text corpora. However, given the limited scope of the experiment, the generalizability of our findings needs to be investigated by further research.

The other three variables that predicted priming reliably were the word frequencies of related primes and targets, as well as the *z*RTs of unrelated primes (with largest beta weights of – .32, – .35, and .25, respectively). Priming was increased following words that occurred less frequently in the corpora. Greater priming was also evident when the targets were low-frequency words. Previous research has also shown that priming effects are stronger for low-frequency words than for high-frequency words (Becker, 1979; Yap, Tse, & Balota, 2009).

Other than their RTs, no unrelated-prime-oriented predictors were significant or accounted for a large amount of variance. The magnitude of influence of unrelated primes has seldom been analyzed in the past (although see Hutchison et al., 2008, who also found significant regression coefficients for the RTs of unrelated primes). More research is needed to explore their effect, and a regression analysis seems to suit this purpose better than a factorial design analysis.

As a variable equivalent to word length in alphabetical languages, the stroke number of neither the primes nor the targets showed any significant or strong impact on priming (beta weights ranging between – .01 and .11). Such results may support the whole-word access theory that Chinese words or, more generally, Chinese characters tend to be processed as holistic perceptual units as opposed to combinations of individual elements (Chialant & Caramazza, 2013; Myers, Huang, & Wang, 2006).

The other variables based on the related primes and targets seemed to exert influences on priming in similar degrees, but in opposite directions. Priming increased with longer target *z*RT_CLP_ but shorter prime *z*RT_CLP_ (the largest beta weights of .23 and – .16, respectively). Targets with more right orthographic neighbors and primes with fewer right orthographic neighbors enhanced priming (the largest beta weights of .17 and – .09, respectively). Priming was greater following primes with fewer left orthographic neighbors, but this tendency was much reduced for targets (the largest beta weights of – .11 and – .03, respectively). Nonetheless, because the beta weights for these variables were not significant, the statistics gave an indication that need to be further investigated.

Among the findings of the present experiment, perhaps the most important, the one that underscores all the others, is what is shared by the information presented in Table 10 and Table 6—there is quite a close correspondence between an association measure’s ability to predict word priming and the degree that it covers the Chen norms. Table 10 shows that the largest beta weights from regression analyses (i.e., at least .31) were derived from the association measures Baroni-Urbani, Jaccard, kappa, log likelihood ratio, Michael, and mutual expectation. Table 6 reveals that the same association measures displayed some of the strongest tendencies (i.e., quotients of 1.57 or below under the paragraph window) to align with the Chen norms. The other two association measures that aligned most strongly with the Chen norms were Gini index and R cost (quotients of 1.49 and 1.45). Although their beta weights from the regression analyses were not as large as .31, they output near-significant coefficients of .22 and .21, which were still greater than the other nonsignificant measures.

Although the explanatory power of the Chen norms for the current priming variances was not very impressive, the fact that the association measures with better predictive ability aligned better with the Chen norms sheds important light on how we could evaluate the norms. It is possible that the association strengths expressed by the norms are generally on the right track, but that a higher level of granularity of the association strengths is required in order to account for the variance in priming. Recall that in comparison to the mostly unique values of the association strengths in the CLAD, normative association strengths tend not to be so rigorously distinguished from each other, due to the relatively small numbers of participants involved in their creation. Because multiple regression analyses are very sensitive to nuance in the magnitude of the numerical variables, we speculate that although the Chen norms and the better-performing association measures are similar in strength, the greater differentiation of strengths yielded by the association measures resulted in their greater ability to explain priming variance.

## Discussion and conclusion

To expand the size or scope of a lexical association database is a goal not unique to this study, but one shared by many of those who constructed traditional association norms. When constructing new norms, researchers often add new stimuli to existing norms. For example, the Palermo and Jenkins’s (1964) association norms are an extension of the Minnesota norms (Jenkins, 1970), and the Edinburgh Word Association Thesaurus (Kiss et al., 1973) contains the stimuli used by Palermo and Jenkins. In addition to incorporating more stimuli, another way to increase norm size is to multiply the set size (i.e., the number of different responses of a stimulus) by using a continuous instead of a discrete association task. In a continuous task, participants are asked to generate more than one response in a sequence, whereas only one response is allowed for discrete associations.

The collection of associative responses for the Chen norms and several frequently cited English association norms were all accomplished through discrete association tasks. By contrast, De Deyne and Storms (2008) constructed word association norms for 1,424 Dutch words using continuous tasks. For each stimulus word, three association responses were gathered per participant, and then they compared the set sizes of the first, second, and third responses. The second and third responses led to a substantial increase of response types: The total amount of response types almost tripled that when calculating only the first response. Furthermore, the first responses were more uniform, whereas the variability increased in the second and third responses. From these results, we can see that continuous association tasks can elicit weak associates, and consequently, it is an effective method for expanding the breadth of associative norms.

Compared to discrete association, continuous association gives subjects much more time to make weaker associations. Doing so not only yields more response types, but also increases the differentiation of association strengths. However, if we were to attempt to explore all the relationships within the mental lexicon with continuous association, we might encounter difficulty removing sources of interference. As De Deyne and Storms (2008) pointed out, continuous association educes chaining and retrieval inhibition (McEvoy & Nelson, 1982). It is inadvisable to allow subjects an unlimited number of responses or to impose no time limit, for otherwise subjects will start responding to their own responses rather than to the target stimulus.

Despite the practical inability of associative norms to cover all words and their lexical relationships, we do not think that corpus-derived lexical association references such as the CLAD can completely replace norms. For example, Joyce (2005) pointed out that word association norms could enhance the ability of bilingual or learner dictionaries to assist in the recollection of terms that would otherwise remain on the tip of the tongue (Brown & McNeill, 1966). When speakers encounter the tip-of-the-tongue phenomenon, they are proactively searching for relevant vocabulary in a way similar to the free association process. Therefore, traditional association norms are likely more appropriate for constructing systems meant to assist users with recollecting words.

Although a full-blown evaluation of the association measures is outside of the scope of this article, it is pertinent to underline the need for the assessment of association measures. We would remind the readers that aside from the challenge of establishing truly objective evaluative criteria, applying association measures itself is a strenuous task. The researchers must first gather an immense amount of linguistic data, preprocess the corpus, compute all the information necessary to calculate association strengths, and only then is it possible to actually apply the measures. In view of these hurdles, we believe the CLAD can provide a wealth of research material for the evaluation of association measures and save future researchers the time and effort normally spent on prep-work.

In this study, we applied 55 statistically oriented association measures. For future research, we are excited to see that new computational algorithms for learning features embedded in language are burgeoning, such as HAL (Lund & Burgess, 1996), LSA (Landauer & Dumais, 1997), BEAGLE (Jones & Mewhort, 2007), Contextual_SOM (Zhao, Li, & Kohonen, 2011), and most recently, Word2Vec (Hsu, Lee, Chang, & Sung, 2018; Mikolov, Chen, Corrado, & Dean, 2013). We look forward to more studies in the future on the design of new methods of measuring the strength of word associations using corpus data, or on how to transform techniques in related fields, such as NLP or AI, into word association measures.

In view of the obstacles to creating more comprehensive association norms, we hope that associations distilled from large text corpora can effectively supplement traditional association norms. Furthermore, for behavior-related systems that require lexical association data, wide applicability cannot be accomplished without the supporting word association database that covers a sufficient amount of association information, and the CLAD or similar large corpus-based association databases could fulfill this role. With the CLAD, we hope to have provided researchers a convenient and comprehensive database that inspires future innovative research.

## Appendix

**Table:** The inventory of word association measures used in this study to compute word association strength

#	Measure	Formula	Reference
1.	Joint probability	*P*(*xy*)	(Giuliano, 1964)
2.	Conditional probability	*P*(*y|x*)	(Gregory, Raymond, Bell, Fosler-Lussier, & Jurafsky, 1999)
3.	Reverse conditional probability	*P*(*x|y*)	(Gregory et al., 1999)
4.	Pointwise mutual information	log$\frac{P(xy)}{P\left(x\ast \right)P\left(\ast y\right)}$	(Church & Hanks, 1990)
5.	Mutual dependency (MD)	log$\frac{P{(xy)}^2}{P\left(x\ast \right)P\left(\ast y\right)}$	(Thanopoulos, Fakotakis, & Kokkinakis, 2002)
6.	Log frequency biased MD	log$\frac{P{(xy)}^2}{P\left(x\ast \right)P\left(\ast y\right)}$+log*P*(*xy*)	(Thanopoulos et al., 2002)
7.	Normalized expectation	$\frac{2f(xy)}{f\left(x\ast \right)+f\left(\ast y\right)}$	(Smadja & McKeown, 1990)
8.	Mutual expectation	$\frac{2f(xy)}{f\left(x\ast \right)+f\left(\ast y\right)}\times P(xy)$	(Dias, Guilloré, Bassano, & Lopes, 2000)
9.	Salience	log$\frac{P(xy)}{P\left(x\ast \right)P\left(\ast y\right)}$log*f*(*xy*)	(Kilgarriff & Tugwell, 2001)
10.	Pearson’s *χ*^**2**^ test	${\sum}_{i,j}\frac{{\left({f}_{ij}-{\hat{f}}_{ij}\right)}^2}{{\hat{f}}_{ij}}$	(Manning & Schütze, 1999)
11.	Fisher’s exact test	$\frac{f\left(x\ast \right)!f\left(\overline{x}\ast \right)!f\left(\ast y\right)!f\left(\ast \overline{y}\right)!}{N!f(xy)!f\left(x\overline{y}\right)!f\left(\overline{x}y\right)!f\left(\overline{x}\overline{y}\right)!}$	(Pedersen, 1996)
12.	*t* test	$\frac{f(xy)-\hat{f}(xy)}{\sqrt{f(xy)\left(1-\left(f(xy)/N\right)\right)}}$	(Church & Hanks, 1990)
13.	*z* score	$\frac{f(xy)-\hat{f}(xy)}{\sqrt{\hat{f}(xy)\left(1-\left(\hat{f}(xy)/N\right)\right)}}$	(Berry-Rogghe, 1973)
14.	Poisson significance	$\frac{\hat{f}(xy)-f(xy)\log \hat{f}(xy)+\log f(xy)!}{\log N}$	(Quasthoff & Wolff, 2002)
15.	Log likelihood ratio	−2${\sum}_{i,j}{f}_{ij}\log \frac{f_{ij}}{{\hat{f}}_{ij}}$	(Dunning, 1993)
16.	Squared log likelihood ratio	−2${\sum}_{i,j}\frac{\log {f}_{ij}^2}{{\hat{f}}_{ij}}$	(Inkpen & Hirst, 2002)
17.	Russel–Rao	$\frac{a}{a+b+c+d}$	(Russel & Rao, 1940)
18.	Sokal–Michener	$\frac{a+d}{a+b+c+d}$	(Sokal & Michener, 1958)
19.	Rogers–Tanimoto	$\frac{a+d}{a+2b+2c+d}$	(Rogers & Tanimoto, 1960)
20.	Hamann	$\frac{\left(a+d\right)-\left(b+c\right)}{a+b+c+d}$	(Hamann, 1961)
21.	Third Sokal–Sneath	$\frac{b+c}{a+d}$	(Sokal & Sneath, 1963)
22.	Jaccard	$\frac{a}{a+b+c}$	(Jaccard, 1912)
23.	First Kulczynski	$\frac{a}{b+c}$	(Kulczynski, 1927)
24.	Second Sokal–Sneath	$\frac{a}{a+2\left(b+c\right)}$	(Sokal & Sneath, 1963)
25.	Second Kulczynski	$\frac{1}{2}\left(\frac{a}{a+b}\right)+\left(\frac{a}{a+c}\right)$	(Kulczynski, 1927)
26.	Fourth Sokal–Sneath	$\frac{1}{4}\left(\frac{a}{a+b}+\frac{a}{a+c}+\frac{d}{d+b}+\frac{d}{d+c}\right)$	(Kulczynski, 1927)
27.	Odds ratio	$\frac{ad}{bc}$	(Tan, Kumar, & Srivastava, 2004)
28.	Yulle’s ω	$\frac{\sqrt{ad}-\sqrt{bc}}{\sqrt{ad}+\sqrt{bc}}$	(Tan et al., 2004)
29.	Yulle’s Q	$\frac{ad- bc}{ad+ bc}$	(Tan et al., 2004)
30.	Driver–Kroeber	$\frac{a}{\sqrt{\left(a+b\right)\left(a+c\right)}}$	(Driver & Kroeber, 1932)
31.	Fifth Sokal–Sneath	$\frac{ad}{\sqrt{\left(a+b\right)\left(a+c\right)\left(d+b\right)\left(d+c\right)}}$	(Sokal & Sneath, 1963)
32.	Pearson	$\frac{ad- bc}{\sqrt{\left(a+b\right)\left(a+c\right)\left(d+b\right)\left(d+c\right)}}$	(Pearson, 1950)
33.	Baroni-Urbani	$\frac{a+\sqrt{ad}}{a+b+c+\sqrt{ad}}$	(Baroni-Urbani & Buser, 1976)
34.	Braun–Blanquet	$\frac{a}{\max \left(a+b,a+c\right)}$	(Braun-Blanquet, 1932)
35.	Simpson	$\frac{a}{\min \left(a+b,a+c\right)}$	(Simpson, 1943)
36.	Michael	$\frac{4\left( ad- bc\right)}{{\left(a+d\right)}^2+{\left(b+c\right)}^2}$	(Michael, 1920)
37.	Mountford	$\frac{2a}{2 bc+ ab+ ac}$	(Kaufman & Rousseeuw, 1990)
38.	Fager	$\frac{a}{\sqrt{\left(a+b\right)\left(a+c\right)}}-\frac{1}{2}\ \max \left(b,c\right)$	(Kaufman & Rousseeuw, 1990)
39.	Unigram subtuples	log$\frac{ad}{bc}-3.29\sqrt{\frac{1}{a}+\frac{1}{b}+\frac{1}{c}+\frac{1}{d}}$	(Blaheta & Johnson, 2001)
40.	U cost	log$\left(1+\frac{\min \left(b,c\right)+a}{\max \left(b,c\right)+a}\right)$	(Tulloss, 1997)
41.	S cost	log${\left(2+\frac{\min \left(b,c\right)}{a+1}\right)}^{-\frac{1}{2}}$	(Tulloss, 1997)
42.	R cost	log$\left(1+\frac{a}{a+b}\right)$log$\left(1+\frac{a}{a+c}\right)$	(Tulloss, 1997)
43.	T combined cost	$\sqrt{U\times S\times R}$	(Tulloss, 1997)
44.	Phi	$\frac{P(xy)-P\left(x\ast \right)P\left(\ast y\right)}{\sqrt{P\left(x\ast \right)P\left(\ast y\right)\left(1-P\left(x\ast \right)\right)\left(1-P\left(\ast y\right)\right)}}$	(Tan et al., 2004)
45.	Kappa	$\frac{P(xy)+P\left(\overline{x}\overline{y}\right)-P\left(x\ast \right)P\left(\ast y\right)-P\left(\overline{x}\ast \right)P\left(\ast \overline{y}\right)}{1-P\left(x\ast \right)P\left(\ast y\right)-P\left(\overline{x}\ast \right)P\left(\ast \overline{y}\right)}$	(Tan et al., 2004)
46.	*J* measure	max[$P(xy)\log \frac{P\left(y|x\right)}{P\left(\ast y\right)}+P\left(x\overline{y}\right)\log \frac{P\left(\overline{y}|x\right)}{P\left(\ast \overline{y}\right)},$ $P(xy)\log \frac{P\left(x|y\right)}{P\left(x\ast \right)}+P\left(\overline{x}y\right)\log \frac{P\left(\overline{x}|y\right)}{P\left(\overline{x}\ast \right)}$]	(Tan et al., 2004)
47.	Gini index	$\max \Big[P\left(x\ast \right)\left(P{\left(y|x\right)}^2+P{\left(\overline{y}|x\right)}^2\right)-P{\left(\ast y\right)}^2$ $+P\left(\overline{x}\ast \right)\left(P{\left(y|\overline{x}\right)}^2+P{\left(\overline{y}|\overline{x}\right)}^2\right)-P{\left(\ast \overline{y}\right)}^2,$ $P\left(\ast y\right)\left(P{\left(x|y\right)}^2+P{\left(\overline{x}|y\right)}^2\right)-P{\left(x\ast \right)}^2$ $+P\left(\ast \overline{y}\right)\left(P{\left(x|\overline{y}\right)}^2+P{\left(\overline{x}|\overline{y}\right)}^2\right)-P{\left(\overline{x}\ast \right)}^2$]	(Tan et al., 2004)
48.	Confidence	max[*P*(*y*| *x*), *P*(*x*| *y*)]	(Clark & Boswell, 1991)
49.	Laplace	$\max \left[\frac{NP(xy)+1}{NP\left(x\ast \right)+2},\frac{NP(xy)+1}{NP\left(\ast y\right)+2}\right]$	(Clark & Boswell, 1991)
50.	Conviction	$\max \left[\frac{P\left(x\ast \right)P\left(\ast \overline{y}\right)}{P\left(x\overline{y}\right)},\frac{P\left(\overline{x}\ast \right)P\left(\ast y\right)}{P\left(\overline{x}y\right)}\right]$	(Brin, Motwani, Ullman, & Tsur, 1997)
51.	Piatetsky-Shapiro	*P*(*xy*) − *P*(*x*∗)*P*(∗*y*)	(Piatetsky-Shapiro, 1991)
52.	Certainty factor	$\max \left[\frac{P\left(y|x\right)-P\left(\ast y\right)}{1-P\left(\ast y\right)},\frac{P\left(x|y\right)-P\left(x\ast \right)}{1-P\left(x\ast \right)}\right]$	(Shortliffe & Buchanan, 1975)
53.	Added value	max[*P*(*y*| *x*) − *P*(∗*y*), *P*(*x*| *y*) − *P*(*x*∗)]	(Sahar & Mansour, 1999)
54.	Collective strength	$$ \frac{P(xy)+P\left(\overline{x}\overline{y}\right)}{\begin{array}{c}P\left(x\ast \right)P\left(\ast y\right)+P\left(\overline{x}\ast \right)P\left(\ast \overline{y}\right)\\ {}\end{array}} $$ $\frac{1-P\left(x\ast \right)P\left(\ast y\right)-P\left(\overline{x}\ast \right)P\left(\ast \overline{y}\right)}{1-P(xy)-P\left(\overline{x}\overline{y}\right)}$	(Aggarwal & Yu, 1998)
55.	Klösgen	$\sqrt{P(xy)}\mathrm{Added}\ \mathrm{value}$	(Klösgen, 1992)

Measures that work under the assumption of statistical independence employ both observed frequencies (as shown in Table 1) and expected frequencies $\hat{f\ }$(*xy*) = *f* (*x**) *f* (**y*) ∕ N. The estimated probabilities of (co-)occurrence are expressed by *P*. *P*(*x*|*y*) is interpreted as the probability of word *x* given word *y*.

## Footnotes

1 We should be safe to assume that the different sizes of the stimulus sets can be exempted from being responsible for the variation of coverage rates, because the Chen norms employed 1,200 stimuli, and each set of stimuli contained a relatively large number of words.
